# Unlocking the Environmentally Independent Conductivity of Nanoengineered Cement Composites with Carbon Nanotubes

**DOI:** 10.3390/ma19214672

**Published:** 2026-11-03

**Authors:** Eryk Goldmann, Marcin Górski, Małgorzata Safuta

**Affiliations:** Department of Structural Engineering, Faculty of Civil Engineering, Silesian University of Technology, Akademicka 5, 44-100 Gliwice, Poland; eryk.goldmann@polsl.pl (E.G.); marcin.gorski@polsl.pl (M.G.)

**Keywords:** cement nanocomposites, carbon nanotubes, electrochemical impedance spectroscopy, electrical conductivity, temperature, humidity

## Abstract

**Highlights:**

- The electrical response of cement composites was measured under varying levels of humidity and varying temperatures at constant humidity.
- The influence of humidity and temperature was clearly separated.
- The influence of temperature was significant in under-percolated samples.

**Abstract:**

Cement nanocomposites stand out as true multifunctional materials, offering not only increased strength or durability, but also advanced capabilities such as real-time strain and crack sensing, on-demand heat generation and energy harvesting. However, smart cement nanocomposites remain vulnerable to their surroundings: shifts in temperature and humidity can destabilize their electrical response and undermine their reliability as strain sensors. To address this challenge, we introduce cement composites incorporating multiwalled carbon nanotubes that deliver a stable electrical response under various working conditions. We compared the electrical response of cement mortars with carbon nanotubes using electrochemical impedance spectroscopy, capturing their behavior across a wide alternating current frequency range under different humidity conditions and, separately, under various temperatures with negligible humidity differences. Two compositions of mortars were tested with carbon nanotube dosages ranging from 0.05 wt.% to 1.0 wt.%. Three levels of humidity, including full saturation, ca. 50% saturation, and fully dried samples, as well as temperatures between 5 °C and 80 °C, were tested. The results indicate that the dosages of carbon nanotubes above the percolation threshold, i.e., 1.0 wt.%, result in high conductivity of cement composites that are stable even under changing humidity and temperature, therefore showing huge potential in their application as next-generation structural sensors, tailored to operate in real-world environments.

## 1. Introduction

Concrete is the most widely used construction material on Earth, with its global production reaching around 30 billion tons per year [1]. Nevertheless, concrete’s widespread use comes with a significant environmental burden. The production of cement, the key ingredient of concrete, is responsible for more than 7% of annual anthropogenic greenhouse gas emissions [2,3] as well as high consumption of water and natural aggregates, leading to resource depletion [4]. Due to the inherent brittle nature of concrete, its low tensile strength [5], and its high vulnerability to corrosive media, which cause severe deterioration of structures, there is a need for effective long-term structural monitoring and cost-effective retrofitting solutions [6].

Nanotechnology offers a possibility to address both challenges by improving the performance of concrete as well as developing new, functional materials, including conductive and self-sensing cement composites [7,8]. In particular, carbon nanotubes (CNTs), cylindrical carbon structures first identified in the 1990s [9], whose atomic arrangement results in an exceptional combination of mechanical strength, electrical conductivity, and thermal conductivity, are among the most promising carbon nanomaterials applied in concrete technology.

The addition of carbon nanotubes into naturally dielectric cement materials allows for the formation of conductive pathways within the matrix, thus leading to the measurable, high electrical conductivity of the composite. CNT-based cement composites represent an emerging class of novel, functional materials, connecting standard load-bearing or retrofitting roles with the ability to measure strains and cracks [10,11,12,13,14,15,16,17,18], generate heat [19,20,21], or harvest energy [22,23,24,25]. However, despite their powerful capabilities, smart cement materials are susceptible to various environmental conditions, particularly significant variations in temperature and humidity. This can result in unstable electrical responses and therefore compromise their reliability as strain sensors, since such inaccuracies in measurements pose a risk of misdiagnosis of the structural behavior. Therefore, gaining a deep understanding of the influence of environmental factors on the stability of electrical conductivity of cement nanocomposites is essential for the field of concrete nanotechnology.

Notably, multiple attempts were made to explain and describe mechanisms of electrical conductivity in cement composites with the addition of carbon nanomaterials subjected to various humidity conditions. A study of the influence of moisture content on the electrical properties of cement nanocomposites was undertaken by Piana et al. [26], who underlined an important influence of moisture on the determination of the percolation threshold, i.e., the optimal amount of the nanofiller required to obtain a stable electrical response. The differences between conductivity in the dry and wet states were up to several orders of magnitude. Such dependence was also described by Hong [27], who determined the percolation threshold at which the influence of humidity was negated by the presence of 0.5 wt.% of carbon nanotubes. For samples with 0.5 wt.% of CNTs, the variation in resistivity was approximately 2.5 times smaller as the relative internal humidity dropped from 100% to 50%. Similarly, Jang et al. [28] concluded that the water ingress and water absorption of cement composites with the addition of CNTs have a significant effect on the resistivity below the percolation threshold, which was found to be 0.2 wt.% of CNTs.

Moreover, it is worth pointing out that the electrical behavior of cement composites incorporating CNTs under various temperatures, to the best of our knowledge, remains relatively unexplored. Nevertheless, various studies on other functional fillers, such as carbon nanofibers [29], brass fibers [30], or a mix of graphite and CNTs [31], have revealed that the electrical and piezoresistive measurements are highly sensitive to temperatures below and above 20 °C.

Therefore, to fill this gap, we present novel, CNT-based cement composites featuring a stable electrical response under various humidity and temperature conditions. The electrical performance of cement mortars containing commercially available multiwalled carbon nanotubes (MWCNTs) was evaluated using electrochemical impedance spectroscopy, allowing us to obtain precise control over the electrical response of cement nanocomposites within a wide range of alternating current frequencies and thus to identify key components which contribute to the overall electrical conductivity of MWCNT-based cement mortars. The influence of humidity was clearly separated from the temperature so that both factors could be assessed independently from one another. Such an approach has not been commonly used in the literature, where the influence of humidity has predominantly been tested and where, in the investigation of the influence of temperature, humidity has not been carefully monitored. A wide range of multiwalled carbon nanotube contents, both below and at the percolation threshold, and two compositions regarding cement-to-aggregate ratio were thoroughly tested. The results show that cement composites incorporating CNTs hold immense potential for the next generation of structural sensors, acting independently of environmental conditions.

## 2. Materials and Methods

### 2.1. Materials

Cement mortars were composed of cement, standard sand according to PN-EN 196-1 standard [32], water suspension of multiwalled carbon nanotubes, tap water, and two types of superplasticizer: liquid, naphthalene-based superplasticizer produced by Mapei, used to disperse carbon nanotubes in water, and polycarboxylate-ether-based superplasticizer in powder form, supplied by BASF to provide proper flowability of the fresh mix. The cement used was ordinary Portland cement CEM I 42.5R supplied by Heidelberg Materials (Heidelberg, Germany). Chemical composition, phase composition, and physical properties of cement are given in Tables S1–S3, respectively. All presented values are declared by the manufacturer and are based on laboratory quality tests.

Commercially available multiwalled carbon nanotubes, NANOCYL NC7000, produced by Nanocyl Belgium (Sambreville, Belgium), were used within this study as a nanomaterial. As declared by the producer, MWCNTs feature an average length of 1.5 µm, an average diameter of 9.5 nm, and 90% purity (Table 1).

### 2.2. Preparation of MWCNTs Dispersion

To prepare MWCNT dispersion, a specified amount of carbon nanotubes was added to 100 g (in case of samples with less than 0.5 wt.% of a nanomaterial) or 200 g (in other cases) of demineralized water with a naphthalene-based superplasticizer. The constant superplasticizer-to-MWCNTs ratio of 5:1 was kept for all samples. The suspension was then ultrasonically mixed using the Hielscher Ultrasonics UP200St ultrasonic homogenizer, which has a power of 200 W. Sonication was divided into 30 s intervals with a 10 s pause in between to avoid water evaporation while the container was kept in an ice bath. All dispersions were sonicated for 30 min excluding breaks.

### 2.3. Preparation of Cement Mortars

Cement mortars were prepared following the protocol from PN-EN 196-1 standard [32]. Two main compositions (Table 2) with different cement-to-sand ratios were chosen to improve the flowability. First, series C was manufactured with the standard proportion of components, i.e., a cement-to-sand ratio of 1:3. In these samples the addition of PCE superplasticizer was necessary to ensure proper consistency and workability of mortars. One reference mortar (without the addition of MWCNTs and PCE) and four mortars with MWCNTs dosages of 0.05 wt.%, 0.1 wt.%, 0.2 wt.% and 0.5 wt.% (solid dosage by weight of cement) were prepared. The ratios of ingredients and sonication parameters are based on our previous work [34], in which we have investigated in detail the influence of MWCNT suspension on rheological parameters of mortars and obtained optimal compositions for the tested mortars. UV-vis analysis of tested suspensions and the optimal sonication time were also included in our other work [35]. Second, to exclude the need for PCE use, series 2C was prepared with the cement-to-sand ratio of 1:2. Similarly, one reference mix and five mortars with MWCNTs addition (up to 1.0 wt.%) were manufactured. Notably, in both series the water-to-cement ratio was kept constant at 0.5, and the water used for the preparation of the MWCNTs dispersions was included in this ratio.

Mortars were cast into 4 cm × 4 cm × 16 cm steel molds, covered and cured in a high-humidity environment for 24 h before demolding. Three samples for each mix were prepared. Copper mesh electrodes were embedded in each sample approximately 2 cm from each end. Each electrode had a height of 6 cm and a width of 3 cm. The thickness of the copper wire was 1 mm. Three vertical and six horizontal, evenly spaced parts of wire, were used for each electrode. Drawing from the authors’ previous experiments with different electrode materials, the effect of electrode on the results was considered low and any discrepancies in the results were carefully investigated. After demolding, the samples were cured for 28 days in water.

### 2.4. Electrochemical Impedance Spectroscopy Measurements

Electrical measurements were conducted in a two-probe layout. Samples were tested using a Gamry Reference 620 potentiostat (Warsaw, Poland) operating at an alternating current (AC) frequency of 1 Hz–2.5 MHz and 50 mV of voltage. The results were then fitted using Gamry Echem Analyst 2 software. In some cases, to achieve a higher-quality fit, measurements within the frequency range of 10 Hz–2.5 MHz have been considered to reduce the electrodes’ effect.

Two EIS testing protocols were applied to cement mortars: (1) under varying humidity and constant temperature conditions and (2) under varying temperature with negligible humidity variations.

To assess the influence of humidity, samples were first saturated in water until a constant mass was achieved, i.e., when the difference in mass after 24 h was lower than 0.1%. Samples were then gently wiped of excess water on the surface, and EIS measurements were performed. The second round of measurements was done after drying the samples at 50 °C for 48 h. The final measurement was conducted after drying the samples to a constant mass, as described above. The water content of saturated C and 2C samples amounted to 7.5–8.6% and 10.25–11.30%, respectively, while after 48 h of drying it decreased to 2.1–2.7% and 2.4–4.3%, respectively.

To investigate the influence of temperature on the electrical response of cement mortars, the samples were again saturated with water and placed in a water bath. Mortars were kept in a plastic container on supports with a few centimeters of water under the samples to provide high moisture conditions. The container was closed and placed in a climate chamber at 80% ± 5% relative humidity and varying temperatures, i.e., 5 °C, 20 °C, 40 °C, 60 °C, and 80 °C. The chamber temperature varied by ±1 °C. The choice of 5 °C was dictated by the possibility of investigating an influence of low temperature without risking ice formation inside the matrix and potential microstructural damage as well as alteration of resistivity measurements. The highest temperature of 80 °C was chosen to investigate the influence of high temperature without risking significant changes in the microstructure caused by decomposition of hydration products, as most of them do not decompose at temperatures under 100 °C. The electrical response of the samples was measured after ca. 72 h of exposure to each temperature, which was required to reach the equilibrium state. Samples were weighed before each EIS measurement, and the temperature was recorded using an infrared thermometer. Mass fluctuations were lower than 1% and therefore they were considered negligible.

## 3. Results and Discussion

### 3.1. EIS Under Varying Humidity Conditions

Due to the high complexity and inhomogeneous microstructure of cement composites, being a combination of various liquid and solid phases, it is very difficult to gain a detailed understanding of their electrical behavior and to predict their conductivity under various conditions. In light of these challenges, EIS stands out as an efficient technique for an in-depth investigation of the conductive paths formed within cement composites, allowing to distinguish resistive and capacitive responses of individual mortar components [36,37].

**Figure 1:**
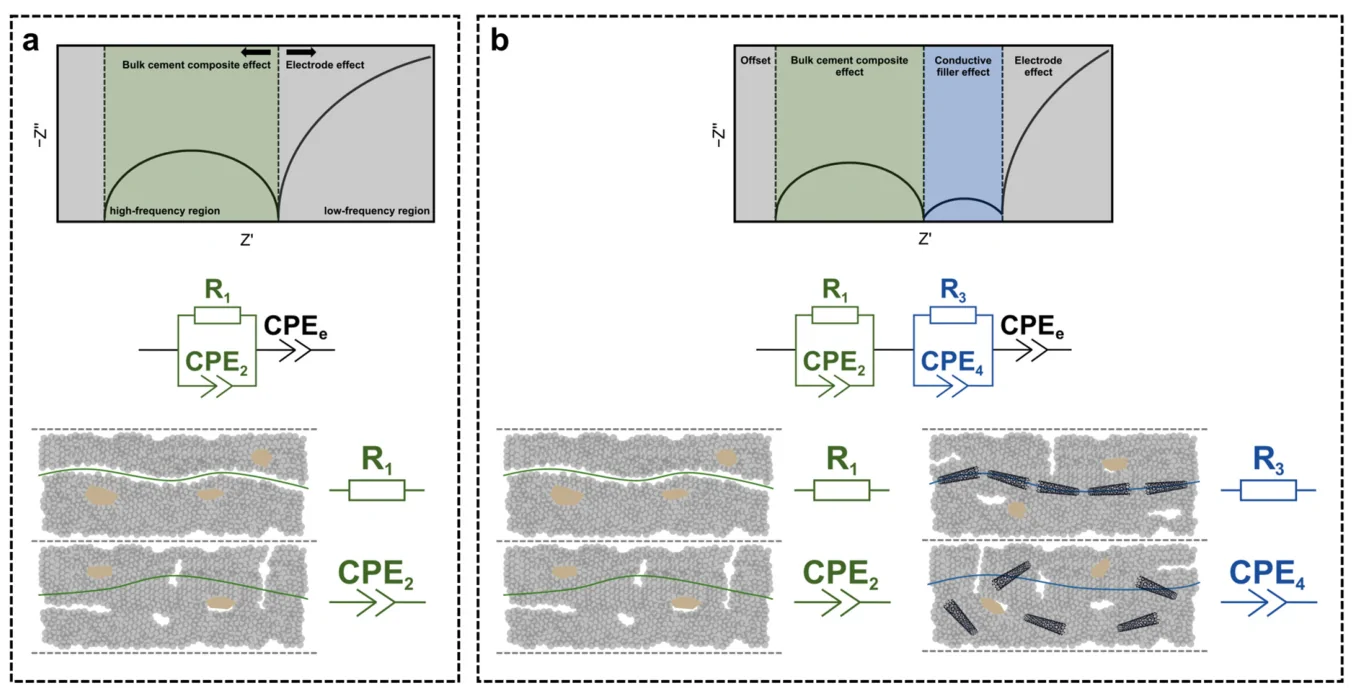
Typical Nyquist plots, conductive paths formed within the structure of the samples, and equivalent electrical circuits applied for the fitting of EIS results for: (**a**) reference mortars and mortars with MWCNTs up to 0.1 wt.%, (**b**) mortars with MWCNTs dosage of 0.5 wt.% and 1 wt.%. R represents a resistor and CPE corresponds to a constant phase element.

Typically, Nyquist plots (the real Z′ vs. the imaginary −Z″ part of impedance) of plain cement composites consist of two characteristic zones (Figure 1a): the high-frequency arc representing the bulk composite effect and the low-frequency line or arc, being a fingerprint of the electrode-specimen interface [38]. The electrical response at the high frequencies can be mimicked with a simple circuit of a resistor (R_1_) connected in parallel with a constant phase element (CPE_2_). A resistor represents the conductive paths formed by continuously connected pores, while CPE corresponds to the pore paths disrupted by solid layers of cement hydrates. Importantly, constant phase elements, i.e., dispersive, frequency-dependent elements, often replace capacitors in circuits established for cement composites, since CPEs are more universal and thus more suitable for the inhomogeneous microstructure of cement composites. The application of CPE gives an inclined semi-circle with the center below the real part axis due to a spread of relaxation times [39]. Moreover, the electrode effect in the low-frequency zone can be represented by another CPE element (CPE_e_).

Interestingly, the high dosage of conductive filler in cement composites results in notable changes in EIS response, i.e., in formation of an additional semi-circle in the intermediate frequency zone (Figure 1b). Therefore, the new Nyquist curve can be simulated with the additional resistor-CPE circuit describing the effect of the functional filler [40,41]. Here, the resistor (R_3_) represents continuous MWCNTs conductive pathways and CPE element (CPE_4_) mimics capacitive paths of solid cement matrix layers with local pores and embedded MWCNTs.

**Figure 2:**
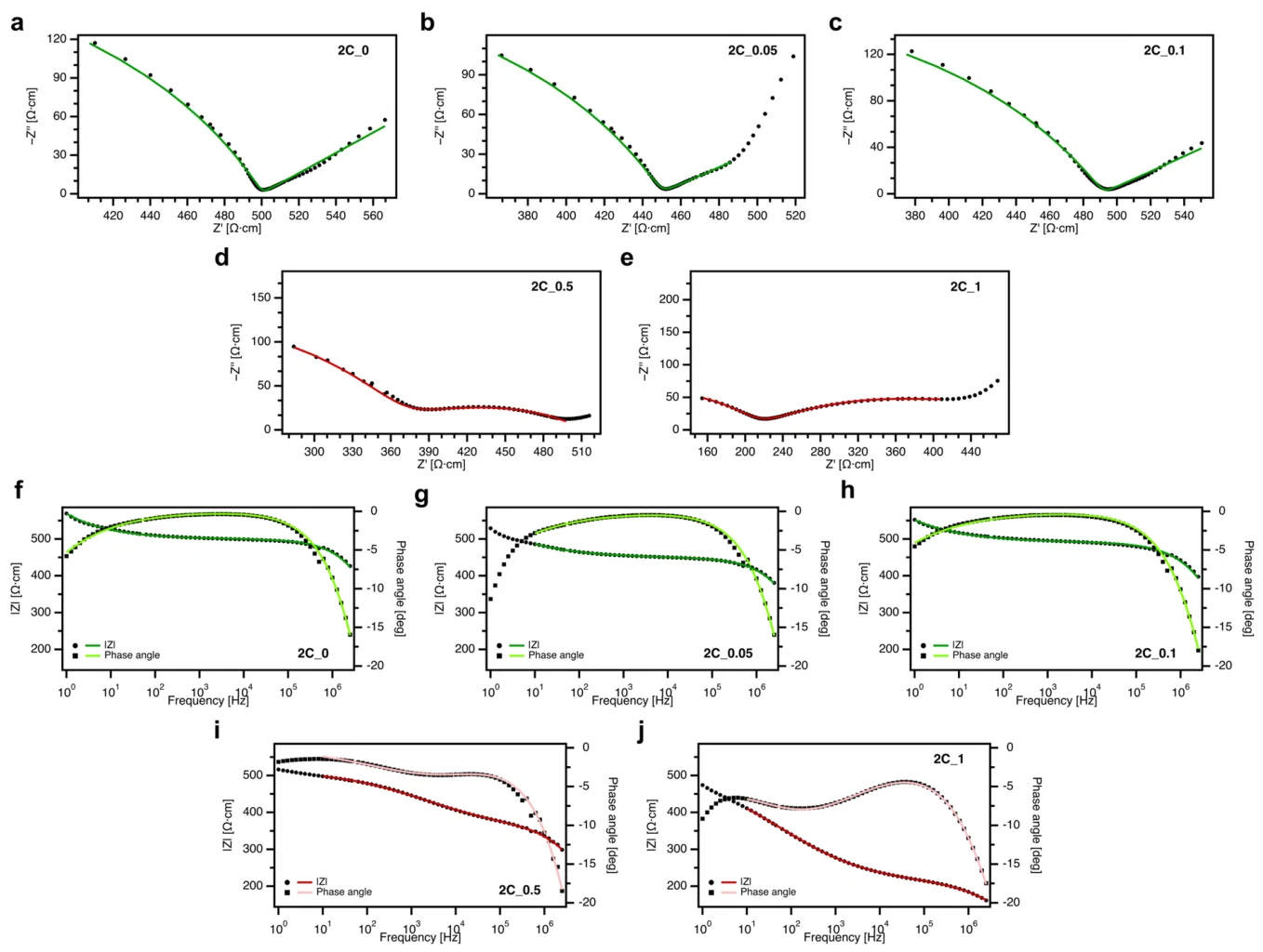
(**a**–**e**) Nyquist and (**f**–**j**) Bode plots for 2C saturated mortar samples. Note: green and red represent the fitting with the equivalent electrical circuits according to Figure 1a,b, respectively. The Nyquist plots were prepared with a constant ratio of Z′ and Z″ axes.

The Nyquist and Bode plots for saturated samples of the 2C series are presented in Figure 2, while the results for the C series can be found in the SI (Figures S1 and S2). The addition of MWCNTs leads to a notable change in the shape of Nyquist plots (Figure 2a–e) obtained for cement mortars: the higher the content of a nanomaterial, the flatter the curve. Moreover, for samples with the highest dosage of MWCNTs, i.e., 0.5 wt.% and 1.0 wt.%, the additional semicircle at the intermediate frequencies is clearly visible, and the zone of electrode-specimen interface becomes smaller, indicating that the impedance response of the composites is dominated by the carbon nanotubes’ conductivity rather than pore solution ionic conductivity. Therefore, the results for reference mortar and mortars with MWCNTs dosage up to 0.1 wt.% were fitted with the circuit presented in Figure 1a, while the circuit from Figure 1b was applied for 0.5 wt.% and 1.0 wt.% samples.

Notably, in both cases (mortars C and 2C), the results for samples with the MWCNTs dosage of 0.2 wt.% are not given due to very high inaccuracies in fitting, which rendered these samples unsuitable for comparison and analysis despite multiple attempts and the use of different equivalent circuits to properly fit the results. This can be attributed to the increasing content of MWCNTs placing 0.2 wt.% mortar in a transition zone between highly resistive composites (reference mortar and mortar with low addition of nanomaterial up to 0.1 wt.%) and conductive mortars (0.5 wt.% and 1.0 wt.%). Most probably, the combination of both behaviors leads to significantly complex electrical response, where the electrical equivalent circuit cannot be clearly established.

In case of Bode plots for saturated samples of 2C series (Figure 2f–j), there were no significant differences in the impedance module between the reference sample and the samples with the lowest carbon nanotubes dosage (up to 0.1 wt.%). For samples with 0.5 wt.% and 1.0 wt.% MWCNTs, a sharper decrease was measured, i.e., the decrease from 426.55 Ω*cm for 2C_0 to 161.66 Ω*cm for 2C_1 (values measured at 2.5 MHz), which can indicate the presence of highly conductive paths inside the matrix. Notably, the typical phase angle curve for reference cement mortar features a characteristic plateau at the intermediate frequency range (ca. 100 Hz–10 kHz) with phase angle values close to zero, an indication of a primarily resistive response. A similar trend was also observed for samples with low dosages of MWCNTs, while the Bode plot obtained for 2C_1 revealed the shift to more capacitive behavior.

**Figure 3:**
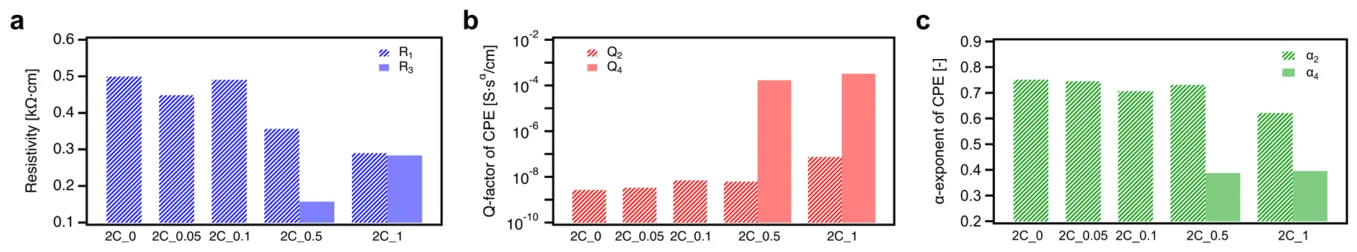
Values of the equivalent circuit components for 2C saturated mortars: (**a**) R_1_ and R_3_, (**b**) Q-factors and (**c**) α-exponents of CPE_2_ and CPE_4_. Note: the values of the components of the equivalent circuits are normalized based on the samples’ geometry and the distance between the electrodes. For the fitting of sample 2C_0.05, the fitting error exceeded the Q_2_ value due to the plot deviation.

Figure 3 presents fitted values for all components of the electrical equivalent circuits for saturated samples of 2C series. The changes in the values of R_1_ and R_3_ resistors illustrate, on the one hand, a possible, slight increase in the porosity of mortars with a high dosage of MWCNTs and, on the other hand, the increasing number of conductive MWCNT pathways created within the matrix. The increasing value of the Q-factor of CPE, as well as the decreasing value of the α-exponent obtained for 2C_1 mortar, further suggests the modification of the pore system and the higher number of MWCNTs embedded within conductive paths.

**Figure 4:**
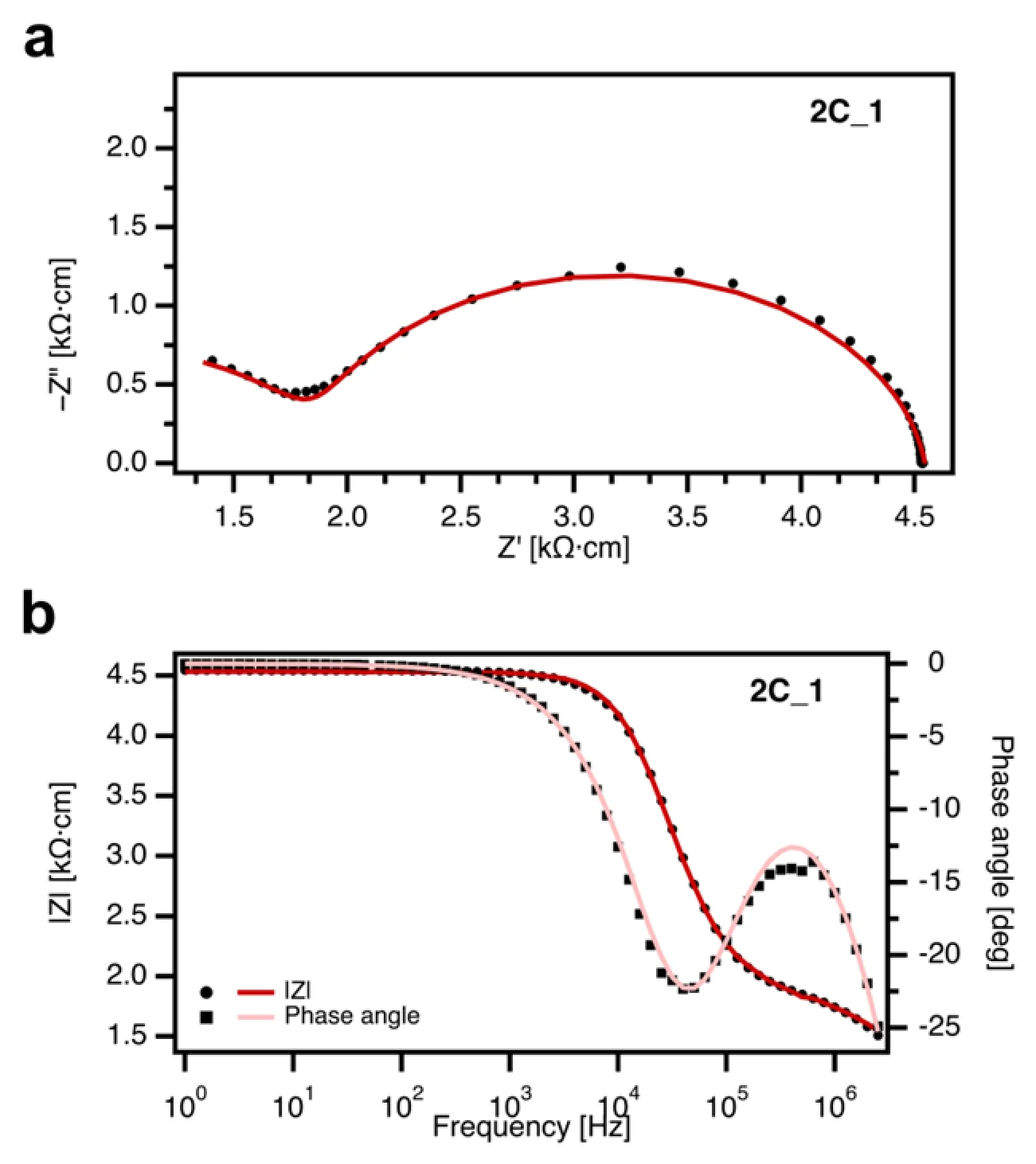
(**a**) Nyquist plot and (**b**) Bode plot for the fully dried 2C_1 mortar sample. Note: red represents the fitting with the equivalent electrical circuits according to Figure 1b.

Furthermore, on the one hand, the measurements of fully dried samples showed that low dosages of MWCNTs are not enough to form continuous conductive paths, effectively compensating for the loss of ionic conductivity of the pore solution, and thus leading to high fitting errors. On the other hand, the measurements of the 2C_1 sample in the dry state (Figure 4) proved the formation of a stable conductive network, indicating that 1.0 wt.% dosage of MWCNTs can be considered as the percolation threshold for the mortars prepared within this study. It is highly visible that the electrical response of the dried 2C_1 sample was dominated by the conductivity of MWCNTs. This observation would fit the definition of percolation threshold proposed by Hong [27] as a dosage that reduces the dependence of electrical response on the sample’s humidity.

### 3.2. Influence of Temperature

**Figure 5:**
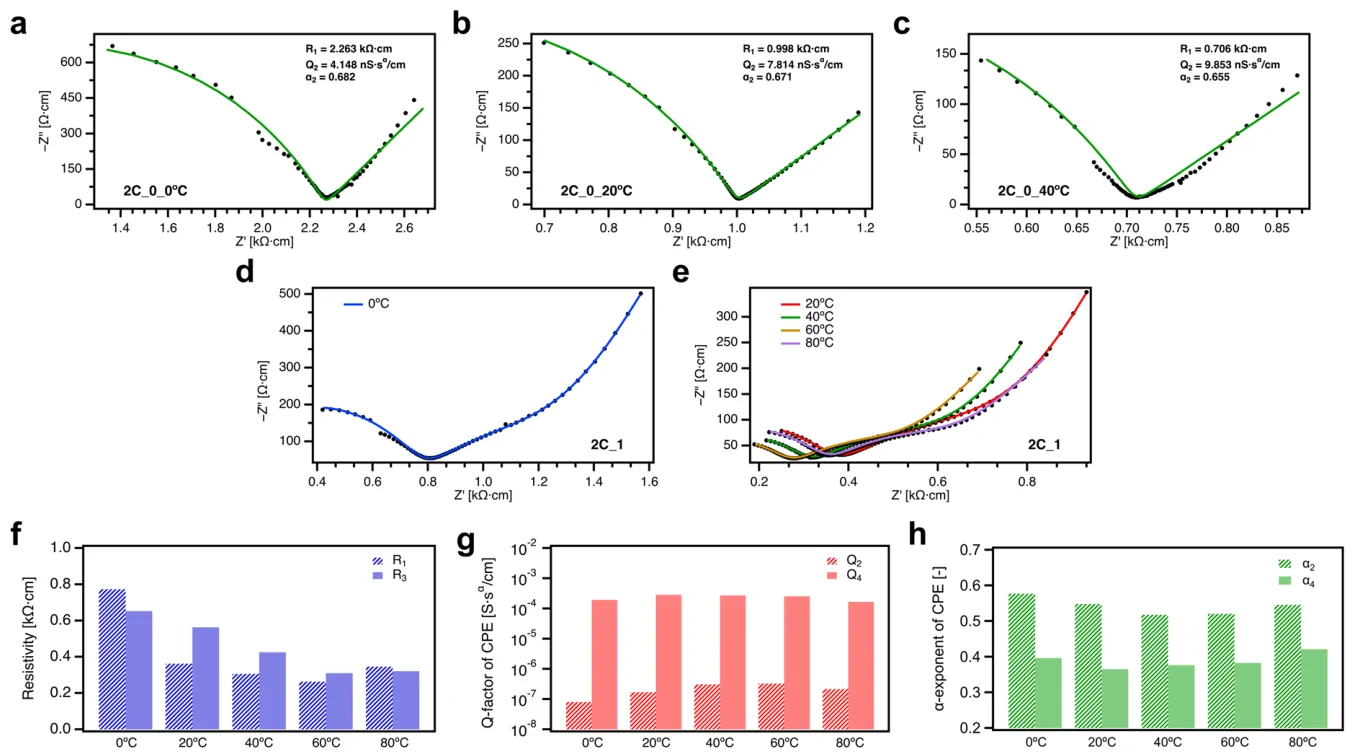
(**a**–**c**) Nyquist plots and the values of equivalent circuit components for the 2C_0 mortar sample kept at the temperature of (**a**) 5 °C, (**b**) 20 °C, and (**c**) 40 °C. Note: green represents the fitting with the equivalent electrical circuits according to Figure 1a. (**d**,**e**) Nyquist plots for 2C_1 mortar sample kept at the temperature of: (**d**) 0 °C, (**e**) 20–80 °C. Note: the Nyquist plots were prepared with a constant ratio of Z′ and Z″ axes. (**f**–**h**) Values of the equivalent circuit components for 2C_1 mortar sample kept at the various temperatures: (**f**) R_1_ and R_3_, (**g**) Q-factors, and (**h**) α-exponents of CPE_2_ and CPE_4_. Note: The values of the components of the equivalent circuits are normalized based on the samples’ geometry and the distance between the electrodes. For the fitting of the measurement at the temperatures of 40 °C and 60 °C, the fitting error exceeded Q_2_ and Q_4_, respectively, due to the plot deviations.

Based on the results discussed above, for further temperature tests, we used samples in a saturated state to minimize the influence of humidity. Figure 5a–c presents the Nyquist plots of the 2C_0 sample kept at 5 °C, 20 °C, and 40 °C, respectively. A clear shift in the plot to lower values of the resistivity can be observed. Notably, higher temperatures, i.e., 60 °C and 80 °C, affected the conduction paths within the composite negatively, and thus further measurements led to the significant dispersion of the results.

The influence of the temperature on the electrical response of sample 2C_1 is highly visible in Figure 5d,e. As the temperature was raised to 60 °C, the resistivity of the samples decreased, which is indicated by the shift in the Nyquist plot to the left. The plot at 80 °C deviates from this trend, as the electrical response falls between the measurements at 20 °C and 40 °C. This reduction in electrical resistivity could be attributed to a higher energy and mobility of ions in the pore solution. As the shape of the graph remains similar, the influence of the temperature differences on the part of the conductivity related to the carbon nanofiller can be considered as negligible. An important deviation from the trend was observed for the measurement done at 80 °C. The rise is resistivity could be by partial migration of water from smaller pore structures which caused local discontinuities in conductive paths. At this temperature range, we would not expect any changes in matrix components as it is too low to cause dissolution of hydration products. Even if some of the Ettringite, which is the least temperature stable, dissolved, ions released would cause a decrease in resistivity, not an increase. Moreover, Bode plots for both sample 2C_0 (Figure S3) and 2C_1 (Figure S4) are given in SI, showing no significant changes in the plots’ shapes.

The values of R_1_ and R_3_ (Figure 5f–h), which correspond to the resistivity of the circuit, follow a linear trend decreasing between 5 °C and 60 °C (from 0.77 kΩ*cm to 0.26 kΩ*cm for R_1_ and from 0.65 kΩ*cm to 0.31 kΩ*cm for R3), while an increase in R_1_ parameter was observed for 80 °C, if compared to 60 °C (up to 0.35 kΩ*cm), with a negligible difference in R_3_ value. The reduction in resistivity for samples after exposure to higher temperatures can be explained by the increased mobility of ions in the pore solution. As samples were saturated, the significance of the ionic conductive path was high; therefore, supplying more energy to ions through heating increased their mobility and, with it, reduced the resistivity. The reduction in R_1_ was also observed for sample 2C_0 as the temperature was rising (Figure 5c), which further backs the possibility of ionic movement being influenced by the higher temperatures. Values of Q-factors did not differ significantly, with only a slight increase between 5 °C and 80 °C.

## 4. Conclusions

Here, we present an intrinsically conductive cement composite incorporating MWCNTs with a relatively unaltered electrical response under various humidity and temperature conditions. Two compositions of cement mortars with the addition of five different dosages of carbon nanotubes were tested in three humidity states at a constant temperature and after exposure to five temperature conditions from 5 °C to 80 °C with negligible variances in humidity. The acquired results showed a key role of the internal humidity of cement nanocomposites for their electrical conductivity, especially for samples under the percolation threshold. The CNTs dosage that allowed for the electrical response being independent of humidity was 1.0 wt.% for series 2C. The influence of temperature was significant in samples with CNTs dosages under the percolation threshold, making it challenging to fit the results with any equivalent circuits. For the sample at the percolation threshold, the effect of temperature was more subtle and evident mostly in the reduction in resistivity with increasing temperature. The increase in temperature lowered the resistivity of cement matrix by ca. 66% up to exposure to 60 °C; however, at 80 °C, there was an increase back by ca. 35%. Notably, the dosage of 0.2 wt.% introduced strong interferences in the EIS measurements, rendering the results not feasible to compare, which might suggest a more complex mechanism appearing at or around this dosage.

Overall, the presented results show that the variations in humidity and temperature do not affect the electrical response of cement nanocomposites with the carbon nanotubes’ dosage above the percolation threshold significantly. The research methodology, which clearly separated the influence of humidity and temperature, ensured that both factors were examined separately and the number of variables was reduced. This allowed for a precise determination of the influence of two of the most common factors affecting concrete structures in their natural working environment, which could also influence the cement-based sensors that monitor these structures. Therefore, CNTs-based cement composites, which demonstrated a stable electrical response under varying conditions, might be used in future works concerning the path to creation of self-sensing cement nanocomposites. Tested properties are one of the factors important for smart cement composites, but they will have to be joined with further piezoresistive tests to fully qualify the tested composites for self-sensing applications. With the influence of temperature and humidity established for the tested samples, future tests will explore other environmental factors that could affect a concrete structure during its lifetime, mainly corrosive factors including chloride and sulfate ingressions and carbonation. Future tests towards self-sensing applications will also have to involve temperature compensation which will be tested alongside other environmental factors.

## Figures and Tables

**Table 1:** Properties of Nanocyl NC7000 [33].

Property	Value
Average diameter, nm	9.5
Average length, μm	1.5
Carbon purity, %	90
Transition Metal oxide, %	<1
Specific area, m^2^/g	250–300
Volume resistivity, Ω·cm	10^−4^

**Table 2:** Composition of tested mortars.

Sample	MWCNT [wt.%]	w/c	Cement-to-Sand Ratio	SP [g]
C_0	0	0.5	1:3	0
C_0.05	0.05			0.5
C_0.1	0.1			0.7
C_0.2	0.2			1.0
C_0.5	0.5			5.0
2C_0	0		1:2	0
2C_0.05	0.05			
2C_0.1	0.1			
2C_0.2	0.2			
2C_0.5	0.5			
2C_1	1.0			

## Data Availability

The data that support the findings of this study are openly available under the CC BY 4.0 license in the open repository “repod.icm.edu.pl” at https://doi.org/10.18150/VTLEES.

## Supplementary Materials

The following supporting information can be downloaded at https://www.mdpi.com/article/10.3390/ma19214672/s1. The file with the following content is available free of charge: The results of Electrochemical Impedance Spectroscopy, chemical and mineral composition of cement, physical properties of cement. Figure S1: Nyquist plots for C saturated mortar samples; Figure S2: Bode plots for C saturated mortar samples; Figure S3: Bode plots for 2C_0 mortar sample kept at the temperatures of 5 °C, 20 °C and 40 °C; Figure S4: Bode plots for 2C_1 mortar sample kept at the temperatures of 5 °C–80 °C; Table S1: Chemical composition of Ordinary Portland cement CEM I 42.5R (OPC); Table S2: Mineral composition of Ordinary Portland cement CEM I 42.5R (OPC); Table S3: Physical properties and strength of Ordinary Portland cement CEM I 42.5R (OPC).
