# Supplementary material for: Unlocking the Environmentally Independent Conductivity of Nanoengineered Cement Composites with Carbon Nanotubes
Source: Materials (Basel). 2026 Nov 3;19(21):4672. doi: 10.3390/ma19214672 (PMC13765157; doi:10.3390/ma19214672)
Supplement: Supplementary file 1 [file materials-19-04672-s001.zip › materials-4644115-supplementary.pdf]

Supplementary Materials

Unlocking the Environmentally Independent Conductivity of Nanoengineered Cement Composites with Carbon Nanotubes

Eryk Goldmann, Marcin Górski and Małgorzata Safuta *

Department of Structural Engineering, Faculty of Civil Engineering, Silesian University of Technology,
Akademicka 5, 44-100 Gliwice, Poland; eryk.goldmann@polsl.pl (E.G.); marcin.gorski@polsl.pl (M.G.)
* Correspondence: malgorzata.safuta@polsl.pl;

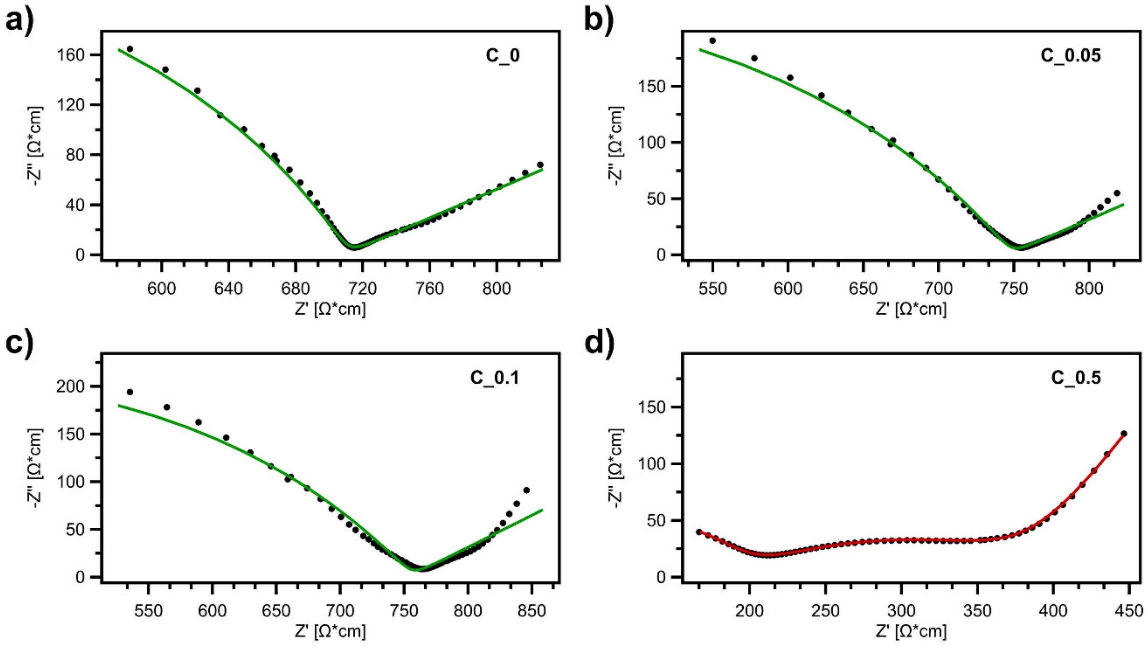

Figure S1. (a)-(d) Nyquist plots for C saturated mortar samples. Note: green and red represent the fitting with the equivalent electrical circuits according to Fig.1a and b, respectively. Note: the Nyquist plots were prepared with a constant ratio of Z' and Z'' axes.

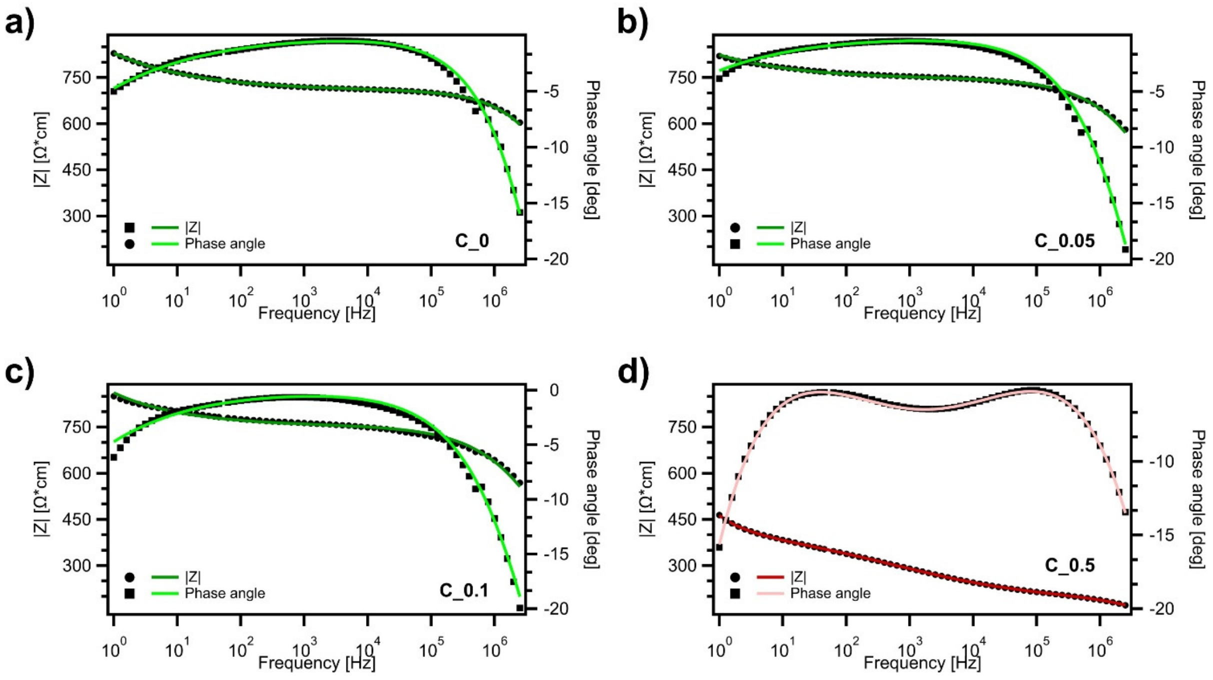

Figure S2. (a)-(d) Bode plots for C saturated mortar samples. Note: green and red represent the fitting with the equivalent electrical circuits according to Fig. 1a and b, respectively.

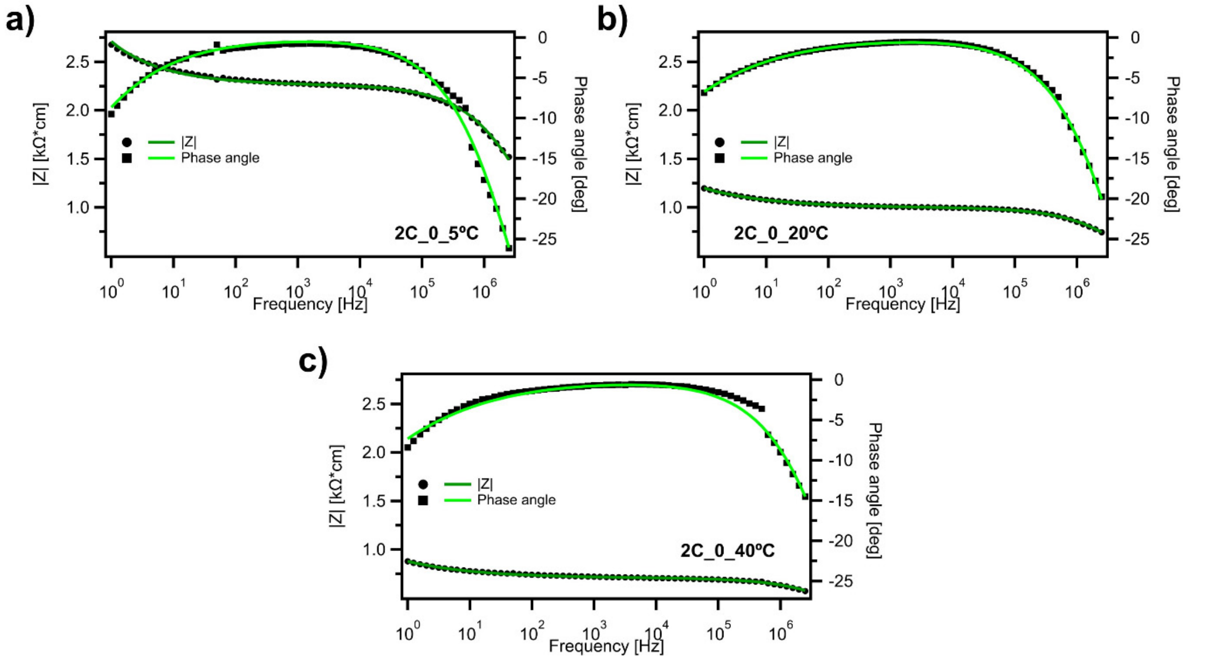

Figure S3. (a)-(c) Bode plots for 2C_0 mortar sample kept at the temperatures of (a) 5°C, (b) 20°C and (c) 40°C. Note: green represents the fitting with the equivalent electrical circuits according to Fig. 1.

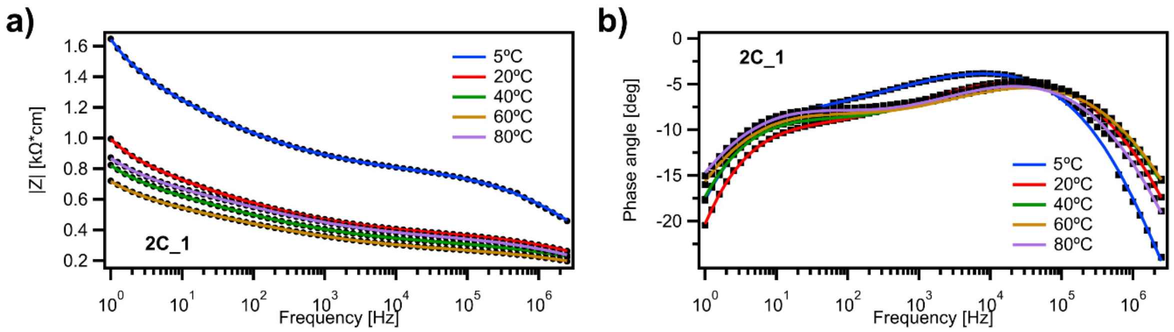

Figure S4. (a) and (b) Bode plots for 2C_1 mortar sample kept at the temperatures of 5°C-80°C: (a) impedance magnitude and (b) phase angle.

Table S1. Chemical composition of Ordinary Portland cement CEM I 42.5R (OPC).

Component	Content, %
Loss on ignition	2.66
Insoluble residue	0.73
SiO_2	20.16
Al_2O_3	5.30
Fe_2O_3	2.69
CaO	63.37
MgO	1.41
SO_3	2.63
Na_2O	0.17
K_2O	0.81
Cl	0.095

Table S2. Mineral composition of Ordinary Portland cement CEM I 42.5R (OPC).

Component	Content, %
Portland clinker	95.7
C_3S	62.8
C_2S	14.9
C_3A	10.4
C_4AF	8.3
Free CaO	1.1
Limestone	4.3

Table S3. Physical properties and strength of Ordinary Portland cement CEM I 42.5R (OPC).

Property	Value
Compressive strength, MPa	
- after 2 days	28.9
- after 28 days	57.8
Water demand, %	27.6
Specific surface, cm^2/g	3510
Initial setting time, min	175
Soundness, mm	0.4
